# Lipid‐Laden Macrophage Index as a Diagnostic Tool for Pediatric Aspiration: A Systematic Review

**DOI:** 10.1002/oto2.33

**Published:** 2023-03-23

**Authors:** Claire M. Lawlor, Sukgi S. Choi

**Affiliations:** ^1^ Department of Otolaryngology Children's National Health System Washington District of Columbia USA; ^2^ Department of Otolaryngology and Communication Enhancement Boston Children's Hospital Boston Massachusetts USA

**Keywords:** aspiration, bronchoalveolar lavage, lipid‐laden macrophages

## Abstract

**Objective:**

Lipid‐laden macrophage index (LLMI) has been proposed as a marker for aspiration on bronchoalveolar lavage. It has also been studied as a marker for gastroesophageal reflux and other pulmonary diseases. This review aims to determine the clinical correlation between LLMI and pediatric aspiration.

**Data Sources:**

PubMed (MeSH search), Scopus, and Cochrane Central Register of Controlled Trials (CENTRAL) portals through December 17th, 2020.

**Review Methods:**

Preferred Reporting Items for Systematic Review and Meta‐Analysis criteria were followed, and a quality assessment of included studies was performed using the Methodological Index for Non‐Randomized Studies. Search criteria included all occurrences in the title or abstract of the terms “pulmonary aspiration” and “alveolar macrophages.”

**Results:**

Five studies describing 720 patients met inclusion, 3 retrospective case‐control studies, and 2 prospective observational studies. Four studies suggested a link between elevated LLMI and aspiration, and 1 found no association. Control groups varied and included healthy nonaspirators to nonaspirators with other pulmonary diseases. Diagnosis of aspiration was not standardized across the studies. Three papers proposed cutoff values for LLMI, all different.

**Conclusion:**

The existing literature indicates that LLMI is not a sensitive or specific marker for aspiration. Further study is needed to define the utility of LLMI in pediatric aspiration.

Pulmonary aspiration in infants and children is the passage of saliva, food, or gastric contents into the airway. Recurrent aspiration may lead to chronic lung diseases including pneumonias and bronchiectasis. In overt aspiration, the laryngeal cough reflex is triggered in an attempt to clear the airway. The absence of a cough or other response to solids or liquids passing through the vocal folds into the airway is termed silent aspiration. The incidence of oropharyngeal aspiration in children presenting with feeding difficulties is reported to be as high as 34%, with 81% of those patients demonstrating silent oropharyngeal aspiration. Silent aspiration is particularly concerning as it lacks obvious clinical signs and may go undiagnosed until chronic lung damage has occurred.^1^ Healthy children may also aspirate but the incidence is unknown due to the lack of reliable and easily testable markers needed to make a diagnosis.^2^ Definitive diagnosis of aspiration is made via videoflouroscopic swallow study (VFSS) or functional endoscopic evaluation of swallowing (FEES).^1^, ^2^ Unfortunately, a single instrumental swallow analysis represents “a snapshot in time,” and may over or under‐diagnose aspiration.

Lipid‐laden macrophages (LLM) in bronchoalveola lavage (BAL) have been investigated as a marker of lung damage in many different clinical conditions, most notably pulmonary aspiration and gastroesophageal reflux in pediatric patients.^3^ The lipid‐laden macrophage index (LLMI) is a measure of the accumulation of lipids within the cytoplasm of alveolar macrophages. It is considered an indication of inflammation though concerns have arisen that it is nonspecific and clinical correlation is inconsistent.^4^ Despite these inconsistencies, the LLMI is still routinely performed in pediatric BAL. If LLMI was determined to be a reliable indicator of pediatric aspiration, it would be beneficial because it can be performed with little risk at the time of endoscopies and BAL. It could serve as a marker for chronic aspiration or microaspiration that may be missed on a single VFSS or FEES. This systematic review seeks to determine the clinical correlation between LLMI and pediatric aspiration.

## Methods

**Figure 1:**
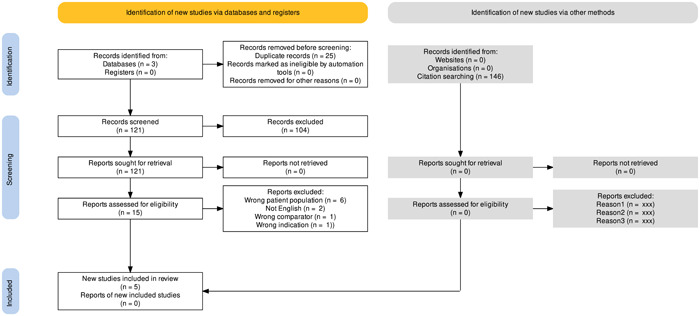
Preferred Reporting Items for Systematic Reviews and Meta‐Analyses flow diagram literature selection process.

A comprehensive review of the English‐language literature was performed using the PubMed (MeSH search), Scopus, and Cochrane Central Register of Controlled Trials (CENTRAL) portals through December 17th, 2020. Search criteria included all occurrences in the title or abstract of the terms “pulmonary aspiration” and “alveolar macrophages.” The article format was limited to clinical trials, clinical studies, randomized controlled trials, controlled trials, validation studies, observational studies, and multicenter studies. Inclusion criteria for the literature search were defined using the Population, Intervention, Control, Outcome, Study Design approach and are detailed in Table 1. A flowchart of the systematic search performed using the Preferred Reporting Items for Systematic Reviews and Meta‐Analyses is given in Figure 1.^5^

**Table 1:** PICOS Inclusion Criteria

Population	Patients under 18 years of age
Intervention	Patients who are clinically or radiographically diagnosed with aspiration
Comparison	Patients who are clinically or radiographically nonaspirators
Outcome	LLMI score
Study design	Case‐control, retrospective cohort

Abbreviations: LLMI, lipid‐laden macrophage index; PICOS, Population, Intervention, Comparison, Outcome, Study Design.

Two reviewers performed an eligibility assessment of the resultant articles in a standardized manner and duplicate publications were excluded. Each abstract was then screened for relevance to the assessment of LLMI in aspirating children. Irrelevant citations and case reports were excluded. The full text of the eligible citations was obtained along with additional records from the reference lists of the published articles. The full‐text articles were reviewed, and noneligible studies were excluded. Studies that examined gastroesophageal reflux and other pulmonary diseases were discarded.

Articles that met the criteria for inclusion were then used for data collection. Data on study design, patient selection and inclusion criteria, diagnosis of aspiration, and quantitative and qualitative LLMI calculations were extracted. Quantitative analysis of pooled data was not performed due to the lack of uniformity in reporting the primary outcomes, adequate control group, or LLMI assay. The risk of bias was assessed with the Methodological Index for Non‐Randomized Studies validated instrument.

## Results

The initial database query identified 146 studies that were screened for relevance to pediatric aspiration and the presence of elevated LLMI on BAL. On initial screening, 104 articles were excluded for lack of relevance, and 25 were duplicates. Fifteen full‐text articles were assessed for eligibility. Manual searching of the 15 full‐text article reference lists did not yield additional eligible studies. In total, 5 articles were included for systematic review. Meta‐analysis was not performed due to the small number of studies that met the criteria and the significant variation between the control groups.

The quality of evidence and risk of bias for each included manuscript is presented in Table 2.^4^, ^6^, ^7^, ^8^, ^9^ There were 3 retrospective case‐control studies and 2 prospective observational studies, all level 3b. Four of the studies were performed at tertiary care centers in the United States and 1 was at a tertiary care center in Mexico.

**Table 2:** Quality of Evidence of Included Studies

Study	Study design	Purpose of study	Source of data collection	Level of evidence	Risk of bias (MINORS)
Reilly 2011	Retrospective case‐control study	Determine LLMI in patients with pulmonary diseases	Tertiary care children's hospital in the US	3b	8/24
Kieran 2010	Retrospective case‐control study	To determine if LLMI was elevated in patients with laryngeal cleft	Tertiary care children's hospital in the US	3b	15/24
Furuya 2007	Prospective observational study	To determine the cutoff value for LLMI in aspirating patients and patients with pulmonary disease	Tertiary care children's hospital in Mexico	3b	9/24
Bauer 1999	Retrospective case‐control study	Assess the sensitivity and sensitivity of LLMI for chronic pulmonary aspiration	Tertiary care children's hospital in the US	3b	9/24
Colombo 1987	Prospective observational study	Evaluate the quantitation of LLMI in patients with a clinical suspicion of aspiration	Tertiary care children's hospital in the US	3b	12/24

Abbreviations: LLMI, lipid‐laden macrophage index; MINORS, Methodological Index for Non‐Randomized Studies; US, United States.

The 5 included manuscripts reported on a total of 720 patients are summarized in Table 3.^4^, ^6^, ^7^, ^8^, ^9^ A total of 263 patients described were diagnosed with “aspiration” and 457 patients acted as controls. One study (Furuya) separated the patients into 3 cohorts: aspirators, those with other pulmonary diseases, and controls. All other studies included only 2 groups, “aspirators” and “non‐aspirators,” which included a combination of asymptomatic patients, patients with other pulmonary diseases, or patients with neurologic or gastroenterology diagnoses.

**Table 3:** Characteristics of Included Studies

Study	Patients with aspiration; other pulmonary diseases; controls	Diagnostic criteria for aspiration	Comparison groups	Mean LLMI + STD aspiration; pulmonary disease; controls	Range LLMI aspiration; pulmonary disease; controls	Cutoff value
Reilly 2011	153; 254; 0	Clinical, some VFSS	Other pulmonary diseases	52.48 ± 33.6; 57.90 ± 40.58; N/A	0‐155; 0‐215; N/A	N/A
Kieran 2010	22; 22; 0	VFSS	Respiratory symptoms undergoing triple endoscopy	62.2 ± 8.6; 49.4 ± 7.81; N/A	N/A	N/A
Furuya 2007	40; 30; 41	Clinical or “radiographic evidence” of aspiration	Pulmonary disease with no signs of aspiration; children without pulmonary pathology	233.3 ± 5.5; 187.8 ± 11.6; 108.5 ± 13.5	145‐305; 50‐291; 5‐248	165
Bauer 1999	26; 87; 0	“Diagnosis in chart” and response to therapy	Pulmonary, GI, neuro, and syndromes with 3 “normal” patients were not analyzed separately	104 ± 62; 44 ± 39; N/A	20‐233; 0‐170; N/A	85
Colombo 1987	22; 23; 0	Clinical	Clinically “non‐aspirators” with 3 patients with CF and moderate pulmonary disease were not analyzed separately	139 ± 46; 21 ± 20; N/A	80‐241; 0‐70	90

Abbreviations: CF, cystic fibrosis; GI, gastroenterology; LLMI, lipid‐laden macrophage index; N/A, not applicable; STD, standard deviation; VFSS, videoflouroscopic swallow study.

All 5 studies employed similar inclusion criteria, including patients 0 to 18 years of age who underwent bronchoscopy with BAL. All BAL samples were analyzed for LLMI according to a protocol described by Colombo and Hallberg in 1987, which was the oldest study included in our analysis.^6^ The alveolar macrophages were scored on a 0 to 4 scale (0: not opacified; 1: up to ¼ opacified; 2: ¼ to ½ opacified; 3: ½ to ¾ opacified; and 4: totally opacified). One hundred consecutive macrophages are scored, and the scores are then totaled for a final result between 0 and 400.

Unfortunately, the diagnosis of aspiration was not standardized among the studies. One study (Kieran) defined aspirators with objective VFSS study results. Three studies (Reilly, Furuya, and Colombo) defined aspiration clinically with only a few patients demonstrating aspiration on VFSS. Clinical signs included episodes of coughing/gagging on multiple occasions while swallowing liquids or solids (Colombo), symptoms of pharyngonasal reflux, cough, choking, cyanosis, respiratory distress, food leakage from a tracheotomy tube, regurgitation, and apnea in patients with risk factors for aspiration (Furuya), and coughing/choking with feeds (Reilly). One study included patients with a diagnosis of “aspiration” documented in the medical record (Bauer).

All studies reported the mean LLMI and the range of LLMI for aspirators and controls. Three studies (Colombo, Bauer, and Furuya) determined a “cut off” value for LLMI based on their data that would raise suspicion for aspiration in a patient. Unfortunately, the cutoff value was not standardized among the studies. Colombo and Hallberg proposed an LLMI of 90/400. Bauer et al proposed 85/400. Furuya et al proposed 165/400.

## Discussion

The aspiration of orogastric contents places a child at risk for pneumonia, choking events, and bronchiectasis. If a child is found to aspirate, they may need feeding therapy, alterations in food delivery, thickening the food bolus, or restricted oral feeds and alternative means of nutrition.^10^ Early and accurate diagnosis of aspiration is essential to minimize these risks and implement only the necessary interventions. LLMI in BAL has been postulated as a diagnostic test for aspiration. When food, saliva, or gastric contents enter the lungs, they are phagocytized by macrophages which can be detected, quantified, and indexed on BAL. Unfortunately, there are many reasons LLMI may be elevated in pediatric patients. LLMI. One known exogenous cause is the use of parenteral nutrition with intravascular intralipid in infants. This results in phagocytosis of the intralipid and the accumulation of LLMs in the alveoli. The breakdown of surfactant, surfactant‐like lipids, and surfactant by‐products can also result in lipids in the airway macrophages. Acute chest syndrome in sickle cell disease, amiodarone use, and aspiration of mineral oil are also documented causes of elevation of LLMI.^11^, ^12^

Unfortunately, the diagnosis of aspiration is not standardized among clinicians nor the authors of the 5 manuscripts in this analysis. Many studies were excluded from this review that attempted to establish a link between LLMI and aspiration, but defined aspirators as patients with clinical or proven gastroesophageal reflux disease (GERD) and presumed gastric contests were aspirated.^13^, ^14^, ^15^ Only 1 of the included studies (Kieran) defined aspiration with VFSS. None used FEES. All others were based on symptoms of aspiration, including coughing/choking with feeds, reflux, choking, cyanosis, apnea, and so forth. These symptoms are nonspecific, and may represent oropharyngeal dysphagia, GERD, eosinophilic esophagitis, or many other feeding difficulties.^10^ Additionally, silent aspirators may not be included in the study population. When the diagnosis and inclusion of “aspirators” are vague, it becomes difficult to determine a true association between the outcome and the disease.

In an effort to determine the utility of the LLMI, it has been investigated as a marker for pulmonary disease including cystic fibrosis (CF), GERD, and aspiration. There are studies demonstrating a link to all of these diseases, and studies demonstrating that the LLMI is nonspecific.^12^, ^13^, ^14^, ^15^, ^16^ In this review, included studies compared “aspirators” to “non‐aspirators.” Only 1 report (Furuya) separated the patients into 3 groups, aspirators, nonaspirating patients with pulmonary disease, and nonaspirating patients without pulmonary disease. The remaining 4 studies included patients with and without other pulmonary diseases in their control groups. Recurrent or chronic pulmonary aspiration is not mutually exclusive from other pulmonary diseases. Some of the patients included as aspirators in these studies had other comorbid pulmonary diseases. Thus though 4 of the 5 included studies did suggest that LLMI was elevated in aspirators as compared to controls, the link is confounded by comorbid disease, especially in light of literature suggesting a link between other pulmonary diseases and LLMI.^12^, ^13^, ^14^, ^15^, ^16^ Reilly et al compared LLMI elevation in aspirators, asthma, CF, recurrent pneumonia, tracheobronchomalacia, and immunocompromised patients and found the LLMI to be a nonspecific marker. Of note, this was the only paper to diagnose aspiration with objective VFSS.

Investigators have sought to determine a “cut off” value of LLMI that would be associated with pulmonary aspiration (or GERD, in excluded studies). Three of the included studies suggested cutoff values ranging from 85 to 165 LLMI.^6^, ^7^, ^8^ The variation in this value brings into question its utility, and there are no studies validating any of the proposed cutoff values.

The LLMI is calculated by pathologists using a protocol described by Colombo and Hallberg. A quantitative tool such as this should have minimal variability to be considered reliable and reproducible. Studies have suggested significant intra‐and interrater variability among pathologists calculating LLMI.^11^, ^12^ Significant variation has been reported between the sensitivity (57%‐100%) and specificity of LLMI (57%‐89%).^15^ This further confounds the utility of the LLMI.

The quality of evidence in this review is relatively poor and the number of patients included in the 5 studies was low at 720. All included studies were retrospective case controls or prospective observational studies, both level 3b. More importantly, the diagnosis of aspiration was nonspecific. Though 4 of 5 studies suggested a link between aspiration and LLMI, the association is weak at best. The ambition of determining a minimally invasive, objective, sensitive, and quantitative tool for the diagnosis of chronic or recurrent aspiration in children is admirable. Future studies with more strict diagnostic criteria, a healthy control group, minimized comorbid pulmonary disease, and a larger sample size may better define the utility of the LLMI in children.

## Conclusion

Four of the 5 included studies suggested a link between chronic or recurrent aspiration in children and an elevated LLMI. Unfortunately, the diagnosis of aspiration was subjective and nonspecific and there was significant variation among control groups, ranging from pulmonary disease to healthy patients. There was wide variation in proposed “cut off” values suggested to diagnose aspiration. Nonincluded studies have also suggested links between LLMI and GERD or other pulmonary diseases including CF. At the present, the existing literature indicates that LLMI is not a sensitive or specific marker for aspiration. Further study is needed to define the utility of LLMI in pediatric aspiration.

## Author Contributions

**Claire M. Lawlor**, study collection and design, data collection, analysis/interpretation of results, manuscript production and revision; **Sukgi S. Choi**, study collection and design, data collection, analysis/interpretation of results, manuscript production and revision.

## Disclosures

### Competing interests

None.

### Sponsorships

None.

### Funding source

No funding or support was obtained.
